# An Unusual Finding: Papillary Tumor of the Pineal Region

**DOI:** 10.7759/cureus.34725

**Published:** 2023-02-07

**Authors:** Grace Kennedy, Arielle Degueure, Min Dai, Areli Cuevas-Ocampo, Octavio Arevalo

**Affiliations:** 1 Radiology, Louisiana State University Health Shreveport, Shreveport, USA; 2 Pathology, Louisiana State University Health Shreveport, Shreveport, USA; 3 Neuropathology, Louisiana State University Health Shreveport, Shreveport, USA; 4 Neuroradiology, Louisiana State University Health Shreveport, Shreveport, USA

**Keywords:** neuroradiology, hydrocephalus, brain tumor, pineal region, papillary tumor

## Abstract

A papillary tumor of the pineal region (PTPR) is a rare tumor of neuroepithelial origin formed from specialized ependymocytes of the subcommissural organ located in the lining of the posterior commissure, not the pineal gland itself. Patients with this type of tumor generally present with nonspecific symptoms secondary to obstructive hydrocephalus such as headache and vision changes. The mean age of patient presentation is 31, with a slight predominance in females. This type of tumor has a high rate of recurrence (56%) following surgical resection. This case study describes the presentation of this uncommon tumor in a 61-year-old woman, including presentation, imaging, surgery, and pathology findings.

## Introduction

A papillary tumor of the pineal region (PTPR) is a rare tumor of neuroepithelial origin formed from specialized ependymocytes of the subcommissural organ located in the lining of the posterior commissure, not the pineal gland itself [1]. The mean age of presentation is 31, with a slight predominance in females [2]. Prior to its distinction by the World Health Organization in 2007, these masses were previously reported as various other types of pineal region tumors [3]. Patients generally present with a headache, vision changes, and hydrocephalus, which often recurs following surgical resection [3,4]. This case of a 61-year-old woman with a history of hydrocephalus presented with confusion, difficulty walking, cognitive changes, and decreased daily activity. After an initial resection of a pineal gland mass, one-year postoperative magnetic resonance imaging (MRI) was obtained. This MRI series demonstrated an intrinsically T1 hyperintense and heterogeneously enhancing mass at the pineal region, internal areas of hemorrhage, calcifications, and hypercellularity on imaging. Repeat surgical excision followed by cytopathologic analysis ultimately led to the identification of a papillary tumor of the pineal region.

This case was previously presented as a poster presentation at the LSU Health Shreveport 2022 Radiology Research Symposium on September 2, 2022.

## Case presentation

Appropriate protocols were followed, and consent from the patient was obtained. This project was approved by the hospital's institutional review board. We present the case of a 61-year-old woman with confusion, difficulty walking, cognitive changes, and decreased daily activity. Imaging from a prior institution demonstrated a hyperdense, partially calcified mass in the pineal region extending into the posterior region of the third ventricle, associated with obstructive hydrocephalus. She underwent a frontal ventriculoperitoneal shunt placement and a subsequent suboccipital craniotomy for subtotal tumor resection. The pathology report described a hypercellular, focally calcified pineal tissue with some corpora arenacea noted. While the report noted no clear neoplasm, a pineocytoma could not be ruled out and clinical follow-up was recommended.

**Figure 1:**
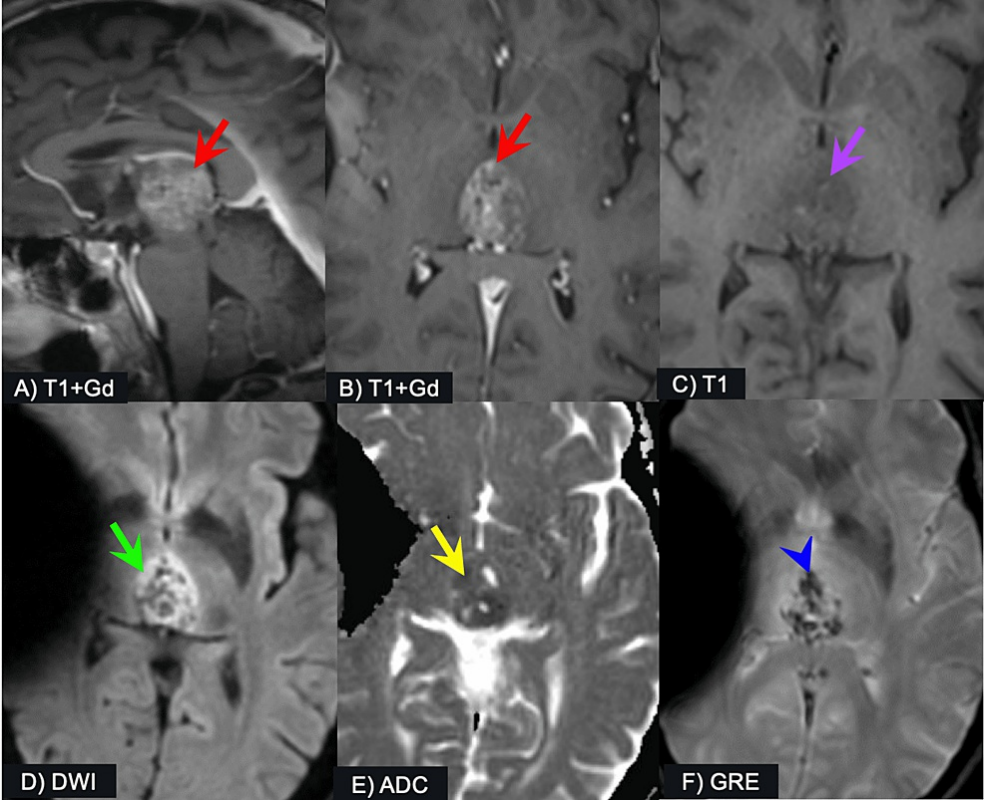
Images from MRI A heterogeneously enhancing and fairly well-circumscribed mass is seen centered at the pineal region (A, B: red arrows), which shows indistinct internal T1-hyperintense foci (C: purple arrow). Diffusion-weighted imaging (DWI) showed areas of restricted diffusion (D: green arrow) and low apparent diffusion coefficient (ADC) values indicating high cellularity (E: yellow arrow). Gradient echo sequences (GRE) revealed internal areas of hemorrhage and calcifications (F: blue arrowhead). Note: artifact present on axial images due to ventriculoperitoneal shunt. MRI - magnetic resonance imaging, Gd - gadolinium contrast

The postoperative MRI of the brain demonstrated a small amount of hemorrhage and a 15 mm residual pineal region tumor. One year postop, our patient had a follow-up contrast-enhanced MRI brain. She had no new relevant symptoms other than headaches, which she described as ‘head pressure’ with accompanying dizziness and nausea several times per week. Physical exam demonstrated nystagmus when looking up. Imaging revealed enlargement of a heterogeneously enhancing, hypercellular mass (2.4 x 2.4 x 2.1 cm) in the pineal region with associated calcification and hemorrhage (Figure 1). The differential diagnosis determined solely from imaging included various types of primary pineal region tumors, such as pineal germinomas and nongerminomatous germ cell tumors, though pineal parenchymal tumor of indeterminate differentiation led to the differential due to the patient’s age, gender, and lesion evolution.

After being reviewed by our tumor board, our patient again underwent a suboccipital craniotomy for a gross total resection via a supracerebellar/infratentorial approach. Pathology identified the resected neoplasm as a papillary tumor of the pineal gland through immunohistochemistry stains and chromosomal microanalysis. The patient received follow-up scans to monitor progression. As demonstrated by MRI at both six months and one year after the second tumor resection, the patient has no signs of tumor recurrence on imaging. This patient did not require chemotherapy or radiation treatment after her second operation. Clinically, the patient has no permanent focal deficits or mental status change at the one-year mark.

## Discussion

This interesting case demonstrates the complex process necessary for a definitive diagnosis of this rare tumor involving imaging, genetic testing, and histopathology. As reported in the literature, PTPRs are generally larger (approximately 3 cm), well-circumscribed masses that sometimes have cystic features [3]. Imaging of these lesions demonstrates contrast enhancement and hypodensity on CT; MRI will reveal enhancement on T1-weighted images with contrast and hyperintensity on T2-weighted sequences [3]. This description matches the image findings of our patient.

Though this patient’s imaging studies followed the known presentation pattern of PTPRs, additional pathology studies were necessary to rule out various other types of more common central nervous system (CNS) tumors that occur in the pineal region. It is important to note that even within these further studies, there can be an overlap between PTPR and other types of lesions. For example, tumors such as pineal parenchymal tumors of intermediate differentiation and ependymomas can look extremely similar to PTPRs histologically [4]. Studies such as immunohistochemical staining (IHC) can further differentiate tissue; PTPRs typically express a variety of cytokeratins like CAM5.2, and epithelial markers such as S100, transthyretin, and vimentin [3]. However, PTPRs and ependymomas can be difficult to distinguish due to their cellular origin as ependymocytes, and thus share many similar markers [1,3]. The combination of multiple tests was important in making a final diagnosis for this patient.

**Figure 2:**
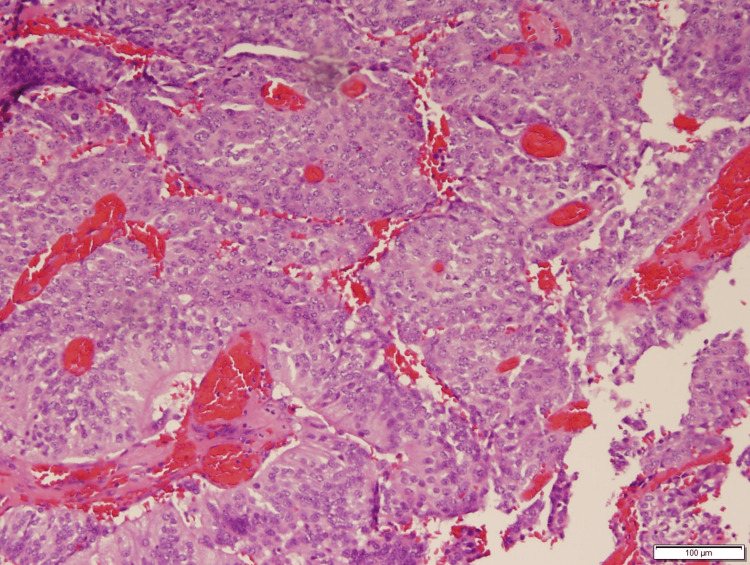
Hematoxylin and eosin stain (H&E) section (x 100) reveals papillary and solid growth pattern, with gland-like perivascular pseudorosettes No evidence of necrosis or microvascular proliferation or brain invasion is seen.

**Figure 3:**
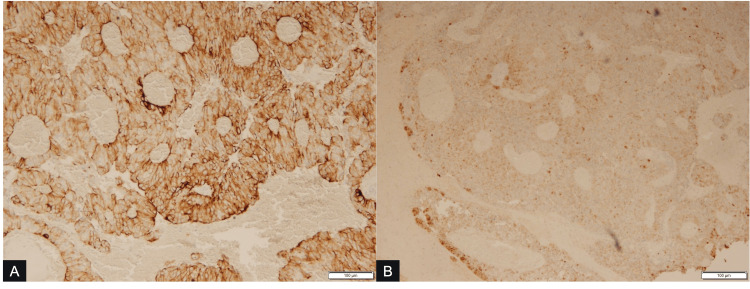
Immunohistochemical (IHC) stain (x 100) demonstrates A) diffuse and strong cytoplasmic membrane positivity for cytokeratin CAM5.2 on tumor cells, with B) patchy positivity for S100 on tumor cells

The mass from this patient underwent additional testing to help make a final diagnosis. Morphologically, the mass demonstrated papillary and solid architecture with rounded epithelioid cells, eosinophilic cytoplasm, round nuclei, granular chromatin, and small nucleoli. The tumor cells formed around fibrovascular cords and form perivascular pseudorosettes (Figure 2). IHC revealed a positive stain for both cytokeratin CAM5.2 and S100 (Figure 3), with a strong, diffuse cytoplasmic membrane stain and patchy positivity for both markers, respectively. Staining was also positive for CK56 and vimentin. This specimen was negative for several cell markers, including GFAP, neurofilament, and synaptophysin.

The chromosomal microarray of the resected tumor was arguably the most helpful study, as it demonstrated the loss of chromosomes 10 and X, and the gain of material on chromosomes 8, 9, and 12. Previous molecular studies have shown the loss of chromosome 10 in 75% of cases of PTPRs, making this feature characteristic of this type of tumor [5]. Additionally, the pattern of genomic alterations present in our patient’s tumor has been reported in prior cases of PTPR and would be an unusual finding for other primary pineal region masses, including pineoblastoma, ependymomas, and primary CNS tumors [6].

## Conclusions

The diagnosis of PTPRs has been increasing since its distinction by the WHO in 2007 as a malignant entity separate from other CNS tumors. While the incidence of these PTPRs may be increasing, the overall prevalence of these tumors remains low. Our case report demonstrates the clinical presentation of a patient with a papillary tumor of the pineal region, the accepted phenomenon of tumor recurrence following the removal of PTPR, and additional knowledge gained through pathology reports such as histological and genetic information. Armed with this understanding of increasing cases, it is important to appreciate that while imaging can help lead to differential diagnoses, additional steps are necessary when making a definitive diagnosis of PTPR.
